# Mechanisms of acquired resistance to afatinib clarified with liquid biopsy

**DOI:** 10.1371/journal.pone.0209384

**Published:** 2018-12-14

**Authors:** Tomomi Nakamura, Chiho Nakashima, Kazutoshi Komiya, Kazuki Kitera, Mitsuharu Hirai, Shinya Kimura, Naoko Aragane

**Affiliations:** 1 Department of Internal Medicine, Division of Hematology, Respiratory Medicine and Oncology, Faculty of Medicine, Saga University, Saga, Japan; 2 ARKRAY Inc., Kyoto, Japan; University of South Alabama Mitchell Cancer Institute, UNITED STATES

## Abstract

Although mechanisms of acquired resistance to 1^st^ and 3^rd^ generation EGFR-TKI continue to be elucidated, there have been few clinical investigations into the mechanisms of acquired resistance to the 2^nd^ generation EGFR-TKI afatinib. We analyzed data from 20 patients with advanced lung adenocarcinoma who acquired resistance to afatinib, including resistance during EGFR-TKI re-challenge. We examined *EGFR* T790M and C797S mutations, *BRAF* V600E mutation, and *MET* amplification with the MBP-QP method and with droplet digital PCR using ctDNA and re-biopsy samples obtained before and after afatinib treatment. Just before afatinib treatment, 15 of the 20 patients were T790M negative and five were positive. Among the T790M negative patients, 40.0% (6/15) became positive at the time of PD under afatinib. In patients positive for T790M, changes in T790M allele frequency were correlated with afatinib treatment efficacy. C797S was not detected in any patients just before afatinib treatment, but it appeared after treatment in three patients, although with very low allele frequency. Two of these three patients, although positive for both C797S and T790M, achieved PR to osimertinib. However, PFS of these patients was somewhat shorter than that of patients positive for T790M only. *BRAF* V600E was detected in one patient at PD under afatinib. *MET* amplification was not detected in this study. T790M is associated with acquired resistance to afatinib, as with 1^st^ generation EGFR-TKI, but with somewhat lower frequency. The influence of C797S on resistance to afatinib is less than that of T790M, but C797S might cause shorter PFS under osimertinib.

## Introduction

Afatinib, a second-generation epidermal growth factor receptor (EGFR) tyrosine kinase inhibitor (TKI), is an irreversible ErbB family inhibitor that binds covalently to cysteine-797 of EGFR and achieves stronger binding ability to ATP binding pocket than 1^st^ generation EGFR-TKIs [1,2]. Phase 3 clinical trials provided evidence that afatinib produces significantly longer progression-free survival (PFS) than gefitinib as a first-line EGFR-TKI treatment for *EGFR* common mutations (exon19 deletion and L858R) in patients with positive non-small cell lung cancer (NSCLC), and higher efficacy in patients with *EGFR* minor mutations such as G719X and L861Q [3,4]. Despite preclinical data suggesting that afatinib might be effective against lung cancer cells harboring secondary T790M mutation [5], a phase 2b/3 randomized trial among lung cancer patients previously treated with EGFR-TKI revealed an overall response rate of only 7% and a PFS of just 3.3 months, indicating that the anti-cancer effect of afatinib on lung cancers containing T790M is clinically insufficient [6]. Osimertinib, the 3^rd^ generation EGFR-TKI, has been developed as an irreversible T790M inhibitor; it binds covalently to cysteine-797 of EGFR as does afatinib. Osimertinib has evidenced significant anti-cancer efficacy in lung cancer patients who are positive for T790M; in particular, PFS after acquired resistance to afatinib was longer than with 1^st^ generation EGFR-TKI [7–10]. It is therefore possible that the mechanism of resistance to afatinib differs from that of 1^st^ generation EGFR-TKIs, and it might lead to higher anti-cancer efficiency with osimertinib treatment.

T790M is the major cause of acquired resistance to 1^st^ generation EGFR-TKI, which occurs in about 50–70% of cases. *MET* amplification, hepatocyte growth factor (HGF) overexpression, and small cell transformation also lead to resistance [11,12]. The mechanisms of resistance to osimertinib have been reported: these include *EGFR* C797S mutation, T790M loss, *EGFR* amplification, *MET* amplification, and *BRAF* V600E mutation [13–19]. On the other hand, there have been few clinical investigations into the mechanisms of acquired resistance to afatinib. In recent studies, T790M was also a cause of acquired resistance to afatinib, but the frequency was lower (36–47%) than with 1^st^ generation EGFR-TKI [20–22], and other modes of acquired resistance have not been clarified. C797S is considered a possible cause of acquired resistance to afatinib because afatinib binds covalently to cysteine-797 of EGFR, as does osimertinib. Nevertheless, unlike with osimertinib, C797S detection during afatinib treatment has rarely been reported.

In this study, we explored mechanisms of acquired resistance to afatinib using circulating tumor DNA (ctDNA) and, when possible, tissue re-biopsy samples. *EGFR* T790M and C797S mutations, *BRAF* V600E mutation, and *MET* amplification were selected for investigation because they are known to lead to acquired resistance to 1^st^ and 3^rd^ generation EGFR-TKI. In addition, we developed a novel, highly sensitive method to detect the C797S mutation in ctDNA. We previously established the mutation-biased PCR and quenching probe (MBP-QP) method to detect T790M and succeeded in detecting T790M in ctDNA with detection rates of 53% and 40%, respectively, among lung cancer patients who acquired resistance to EGFR-TKI in a retrospective study [23] and a multi-centered prospective study [24]. This time, we undertook to develop new detection systems for the two *EGFR* C797S mutations, T2389A and G2390C, using the MBP-QP method. It is necessary to clarify the efficiency with which T790M and other mechanisms of acquired resistance can be detected, to administer appropriate molecular targeted drugs when disease progression occurs under afatinib treatment.

## Patients and methods

### Patient selection

Thirty-six patients with lung adenocarcinoma underwent treatment with afatinib at Saga University Hospital from May 2014 to November 2016 (S1 Fig). Afatinib was administered to sixteen patients as the first line EGFR-TKI treatment, to two patients after discontinuation of the 1^st^ generation EGFR-TKI because of adverse effects, and to eighteen patients after acquired resistance to previous EGFR-TKI as EGFR-TKI re-challenge. Among these 36 patients, we enrolled in this study 20 who developed resistance to afatinib and from whom ctDNA was collected. In the enrolled patients, ctDNA was repeatedly collected throughout the course of treatment, and re-biopsy was performed in eight patients at the time of acquired resistance to afatinib. The study protocol was approved by the Clinical Research Ethics Committee of Saga University. All patients gave informed consent—in accordance with the Declaration of Helsinki—for blood and tissue specimen collection and for genomic testing.

### DNA extraction from plasma samples

Peripheral blood samples were collected into tubes containing 3.8% citric acid. Plasma was immediately separated from blood cells by 3000 rpm centrifugation at 4°C for 20 min. Supernatants were collected and stored at -80°C until assays were performed. From May 2014 to April 2016, DNA was isolated from 200 μl of plasma using a QIAamp DNA mini kit (QIAGEN, Hilden, Germany) according to the manufacturer’s instructions. Subsequently, DNA was isolated from 1000 μl of plasma with a Maxwell RSC cfDNA plasma cartridge (Promega, Mannheim, Germany, product number AS 1480) according to the manufacturers' instructions. All DNA samples were stored at -20°C until further examination.

### Detection of EGFR T790M mutation

T790M in ctDNA was detected by the mutation-biased PCR and quenching probe (MBP-QP) method. This system is fully automated using *i*-densy IS-5320 (ARKRAY Inc., Kyoto, Japan), as described previously [23]. Briefly, MBP-QP consists of two steps: mutation-biased PCR (MBP) and quenching probe (QP) mutation detection. For MBP, the primers for wild-type and mutant were mixed with genomic DNA, which leads to high specificity because each primer can be competitively hybridized to wild type and mutant sequences. In addition, the length of the reverse primer for mutant is longer than that for wild-type, and the annealing temperature is designed to be optimal for mutant primer, resulting in higher amplification efficiency with the mutant sequence. Presence of mutation in amplified sequences was determined by monitoring the fluorescence intensity of a TAMRA-conjugated, guanine-specific quench fluorophore probe (QProbe, J-Bio21, Tokyo, Japan) that is complementary to mutant type sequence containing the mutation part. Fluorescence intensity was measured at different temperatures to identify wild-type and mutant amplicons. T790M from re-biopsy samples was detected by the MBP-QP method and the cobas *EGFR* mutation test (Roche Molecular System, Pleasanton, CA).

### Detection of EGFR C797S mutation

**Fig 1:**
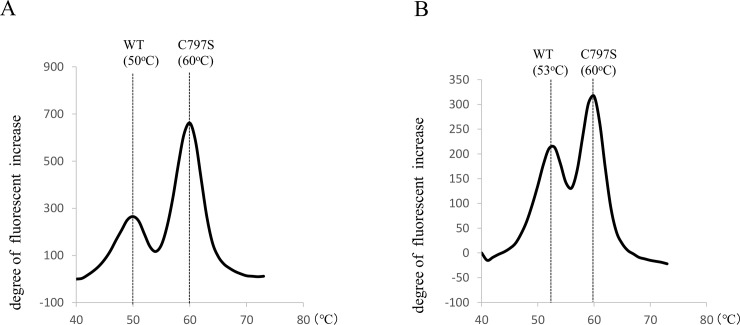
(*A*) A representative result of the MBP-QP method to detect C797S T2389A mutation by using control plasmid. (*B*) A representative result of the MBP-QP method to detect C797S G2390C mutation by using control plasmid.

To detect the *EGFR* C797S mutations, T2389A and G2390C, in both ctDNA and re-biopsy samples, we adapted the MBP-QP method using i-densy (ARKRAY Inc., Kyoto, Japan) (Fig 1A and 1B), as follows. (The MBP-QP method has already been established for detecting the *EGFR* T790M mutation [23].) Volume input to i-densy was 4 μl with each type of DNA sample. At the MBP step, PCR conditions for T2389A were 95°C for 60 s, 62 cycles at 95°C for 1 s, and 62°C for 15 s. PCR conditions for G2390C were 95°C for 60 s, 5 cycles at 95°C for 1 s, 68°C for 15 s, 54 cycles at 95°C for 1 s, and 66°C for 15 s. Primer sets for T2389A were: 5’-GATCAGCAGCTCATCCCCTTCGGCA-3’ for the forward primer of the mutant sequence, 5’-AGTCTAGCTCATCCCCTTCGGCT-3’ for the forward primer of the wild type sequence, and 5’-CCAATATTGTCTTTGTGTTCCCGGACATAGTC-3’ for the reverse primer of the mutant and wild type sequences. Primer sets for G2390C were: 5’-AGCGTGGACAACCCCCACGT -3’ for the forward primer of the mutant and wild type sequences, 5’- TCAGACCGGACATAGTCCAGGAGGG -3’ for the reverse primer of the mutant sequence, and 5’- CTATGCGGACATAGTCCAGGAGGC -3’ for the reverse primer of the wild type sequence. Presence of C797S in the amplified sequences was determined by monitoring the fluorescence intensity of a TAMRA-conjugated, guanine-specific quench fluorophore probe (QProbe, J-Bio21, Tokyo, Japan), which is complementary to C797S: 5’- CTTCGGCAGCCTCC -(TAMRA)-3’ for T2389A and 5’- AGTCCAGGAGGGAGCC -(TAMRA)-3’ for TG2390C. Dissociation temperatures were 60°C for mutant T2389A, 50°C for wild type T2389A, 60°C for mutant G2390C, and 53°C for wild type G2390C (Fig 1A and 1B). The control plasmids were prepared by GenScript USA Inc.: a 300-bp DNA fragment (Accession No. NG_007726.3 167197–167496) was obtained by PCR, purified, and subcloned into the pUC57 vector. The criterion for declaring a sample positive for mutation with the MBP-QP method was that the ratio of areas under mutation and wild-type peaks, multiplied by 100, was 10.9 or greater for T2389A and 6.0 or greater for G2390C. The areas under the mutation peaks were calculated by the “idensy AreaAna” software developed by ARKRAY Inc. Samples were examined with droplet digital PCR (ddPCR) to confirm the results of MBP-QP. Reaction mixtures for ddPCR were assembled from ddPCR supermix for probes (no dUTP) (Bio-Rad, Hercules, CA, USA), LBx probe EGFR C797S multi (riken genesis, Kanagawa, Japan), and pure water. A total of 4 μl of template DNA and 16 μl of reaction mixture was loaded into sample wells. The analysis was performed using a QuantaSoft Droplet Digital PCR QX200 system (version 1.7; Bio-Rad, Hercules, CA, USA) according to the manufacturer’s instructions.

### Detection of EGFR L858R mutation and exon19 deletion

L858R in ctDNA was detected by the MBP-QP method using *i*-densy IS-5320 (ARKRAY Inc., Kyoto, Japan), as described previously [25]. Exon19 deletion in ctDNA was detected by the wild inhibiting PCR and quenching probe (WIP-QP) method using *i*-densy IS-5320 (ARKRAY Inc., Kyoto, Japan), as described previously [26]. Briefly, WIP-QP is a fully automated system with two steps: wild inhibiting PCR (WIP) and quenching probe system (QP). Wild Inhibitor nucleic acid (WI) is complementary to wild type sequence corresponding to the deletion part. WI suppresses amplification of the wild type sequence by binding to wild-type template but not mutant, resulting in preferential amplification of the mutant sequence.

### Detection of BRAF V600E mutation

We used ddPCR to detect *BRAF* V600E mutations in ctDNA and re-biopsy samples. Reaction mixtures for ddPCR were assembled from ddPCR supermix for probes (no dUTP) (Bio-Rad, Hercules, CA, USA), “PrimePCR ddPCR Mutation Assay: BRAF WT for p.V600E, Human” (Bio-Rad, Hercules, CA, USA), “PrimePCR ddPCR Mutation Assay: BRAF p.V600E, Human” (Bio-Rad, Hercules, CA, USA), and pure water. A total of 4 μl of template DNA and 16 μl of reaction mixture was loaded into sample wells and the analysis was performed using the QuantaSoft Droplet Digital PCR QX200 system.

### Determination of MET copy number

We performed ddPCR to determine *MET* copy number in ctDNA and re-biopsy samples. The ddPCR reaction mixture was assembled into a final volume of 20 μl with ddPCR supermix for probes (no dUTP) (Bio-Rad, Hercules, CA, USA), “ddPCR Copy Number Assay: MET, Human” (Bio-Rad, Hercules, CA, USA), “ddPCR Copy Number Assay: RPP30, Human” (Bio-Rad, Hercules, CA, USA) as a reference gene, HaeIII (Takara bio, Kusatsu, Japan), and 7 μl template DNA. Copy number analysis was performed using a QuantaSoft Droplet Digital PCR QX200 system (version 1.7; Bio-Rad, Hercules, CA, USA) according to the manufacturer’s instructions. *MET* copy number was calculated as the ratio of the concentrations of *MET* and *RPP30*. According to a previous report, *MET* copy number > 5.5 by ddPCR and FISH ratio > 2.0 had the highest concordance rate (98%) [27], so, we defined *MET* amplification to exist if *MET* copy number by ddPCR exceeded 5.5.

## Results

### Detection limits for C797S mutation using MBP-QP method

Using the MBP-QP method, we identified the two *EGFR* C797S mutations, T2389A and G2390C, in control plasmid (Fig 1A and 1B). Wild-type and mutant sequences were clearly distinguishable because their melting temperatures (T_m_) differ, as noted in Methods. Detection limit based on serial dilutions of control plasmid was one copy for each mutation. When mutant plasmids and wild type were mixed in different ratios, the detection limit for T2389A was 0.01% mutant plasmids and that for G2390C was 0.05%.

### Patient characteristics

Clinical characteristics of the patients are shown in S1 Table and S2 Table. Ages ranged from 40 to 84 years (median age 63 years); there were 11 females (55.5%) and 11 never-smokers (55.5%). *EGFR* activating mutation was detected in primary tumors of all patients; eight patients (40.0%) had exon19 deletions, eleven (55.5%) had the L858R mutation, and one had the G719A mutation (5.0%). Five patients were given afatinib as the first-line EGFR-TKI treatment. Fifteen patients had previous EGFR-TKI treatment and three of these received afatinib once before the present treatment (S1 Table and S2 Table). Responses to previous EGFR-TKIs and reasons for treatment discontinuation are shown in S2 Table. The best response to afatinib was that of six patients who achieved partial response (PR) (30%) and nine patients who exhibited stable disease (SD) (45%); afatinib was ineffective in five patients (25%). Eight patients underwent re-biopsy at the time of disease progression while under afatinib treatment.

### Detection of EGFR T790M mutation before and after afatinib treatment, and response to afatinib

Table 1 shows T790M detection status in ctDNA before and after afatinib and response to afatinib. T790M was also examined with re-biopsy in patients who experienced progressive disease (PD) under afatinib. Patients 1–7 were treated with afatinib as the first-line treatment or after discontinuation of 1^st^ generation EGFR-TKI due to adverse effects. Patients 8–20 were treated with afatinib after acquired resistance to previous EGFR-TKI treatment, as EGFR-TKI re-challenge. Patients 18, 19, and 20 had a history of afatinib treatment for re-challenge.

**Table 1:** T790M detection with ctDNA and re-biopsy before and after afatinib treatment, and response to afatinib.

Patient*	T790M in ctDNA at PD under previous EGFR-TKI	T790M in ctDNA just before afatinib	Response to afatinib			T790M detection at PD under afatinib	
			ORR	PFS (days)	Duration of treatment (days)	ctDNA	Re-biopsy
1		Negative	PR	575	592	Negative	Negative
2		Negative	PR	120	154	Negative	NE
3		Negative	PR	193	568	Negative	NE
4		Negative	PR	264	362	Negative	NE
5		Negative	SD	358	600	Positive (310)	Positive (274)
6		Negative	SD	88	92	Negative	NE
7		Negative	SD	211	339	Negative	NE
8	Negative	Positive (26.0)	PR	493	566	Positive (24.8)	Negative
9	Positive (25.4) ^†^	Negative	PR	165	206	Positive (15.9)	NE
10	NE	Positive (24.1)	SD	328	609	Positive (22.4)	Positive (268)
11	NE	Positive (11.9)	SD	89	160	Positive (123)	Positive (325)
12	Negative	Negative	SD	98	159	Positive (8.7)	NE
13	Negative	Negative	SD	75	210	Positive (23.9)	NE
14	Positive (22.6)	Negative	SD	131	227	Negative	Positive (147)
15	NE	Negative	SD	119	125	Negative	NE
16	Positive (35.3)	Positive (24.6)	PD	56	343	Positive (63.4)	LN:Positive (42.6)
							Liver: Negative
17	NE	Positive (8.1)	PD	14	54	Positive (67.0)	NE
18	Positive (15.9)	Negative	PD	90	177	Positive (204)	Positive (489)
19	Positive (15.9)	Negative	PD	34	NE	Negative	NE
20	Positive (8.7)	Negative	PD	45	NE	NE	NE

*Patients 1–7 were treated with afatinib as first-line treatment or after discontinuation of 1^st^ generation EGFR-TKI due to adverse effects. Patients 8–20 were treated with afatinib after acquired resistance to previous EGFR-TKI, as EGFR-TKI re-challenge.

^†^Number in parentheses is area under mutation peak of T790M by the MBP-QP method.

Abbreviations: PR, partial response; SD, stable disease; PD, progressive disease

NE, not evaluated; ORR, overall response rate; PFS, progression-free survival

T790M was not detected in ctDNA before afatinib treatment in patients 1–7, and all of these patients achieved PR or SD under afatinib. When tumor progression was detected under afatinib, six patients (all except patient 6) met Jackman’s criteria for acquired resistance to first line EGFR-TKI treatment (Table 1) [28]. T790M in ctDNA turned to positive in one patient after PD under afatinib, and T790M was also detected with re-biopsy collected at the same time from that patient.

Among thirteen patients, numbers 8–20, T790M had already been detected in six patients at PD under previous EGFR-TKI treatment, and thereafter chemotherapy was performed for these patients. T790M disappeared just before afatinib treatment at PD under chemotherapy in five of these patients (patients 9, 14, 18, 19, and 20), and it was positive at PD under afatinib in two patients. T790M was not detected at PD to previous EGFR-TKI in three patients (patients 8, 12, and 13), and in one patient (patient 8) T790M became positive just before afatinib treatment, concomitant with tumor progression during the period with no treatment after previous treatment with EGFR-TKI. When afatinib treatment was started, osimertinib had not yet been approved, so afatinib, which demonstrated suggestive efficacy against T790M positive lung cancer cells in preclinical data, was selected for the patients who had T790M detected just before afatinib treatment.

**Fig 2:**
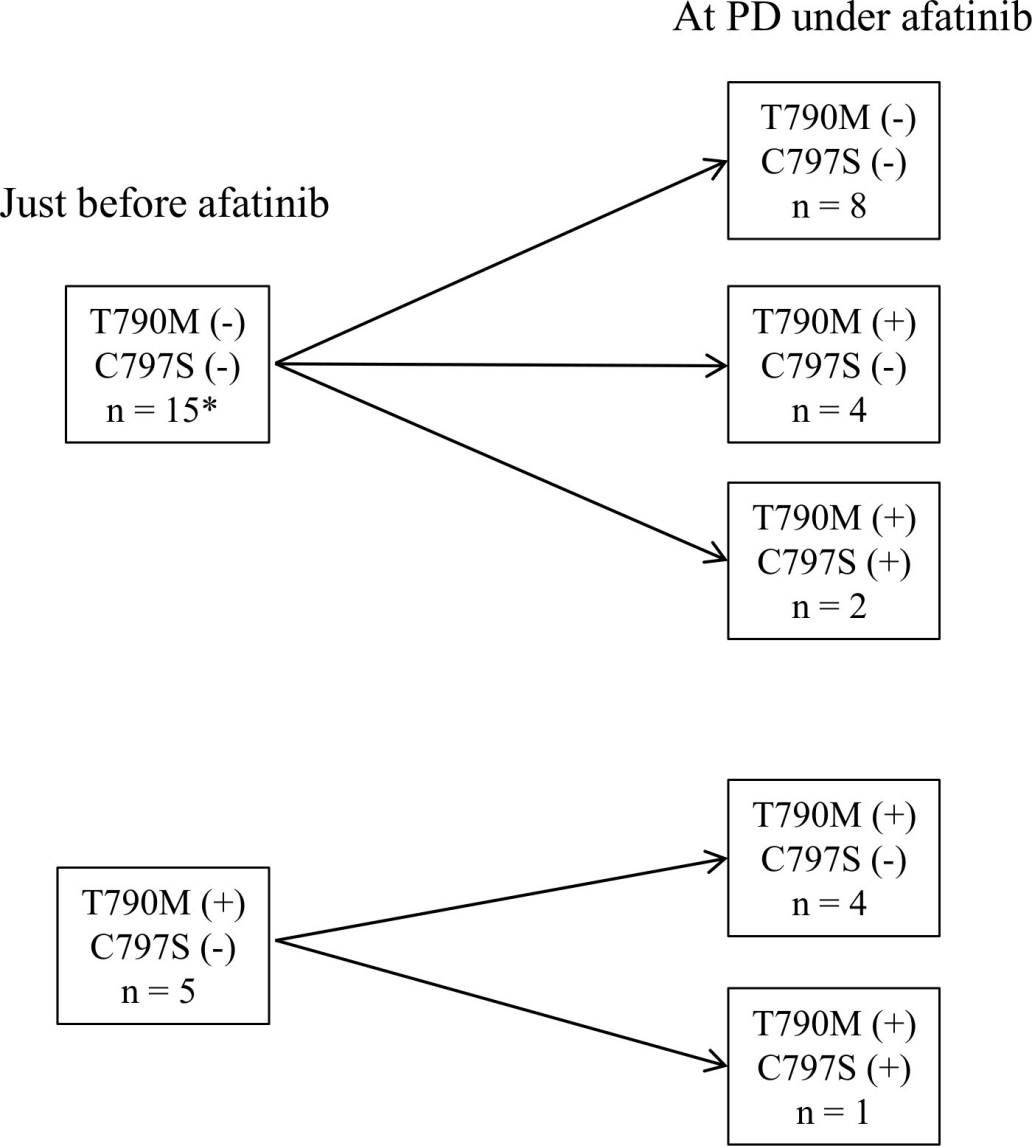
Change of detection of T790M and C797S just before afatinib treatment and at PD under afatinib. *Neither ctDNA nor re-biopsy samples were obtained at PD under afatinib.

In the patients who were treated with afatinib as EGFR-TKI re-challenge, two patients achieved PR and six patients had SD. The PFS of SD patients was shorter than that of patients who were treated with afatinib as first line treatment, but almost all patients continued afatinib after RECIST PD by physician’s decision. Duration of afatinib treatment was nearly five to six months in many patients (Table 1). Afatinib treatment resulted in one PR and two SD among five patients in whom T790M was detected just before treatment. In all of these patients, T790M was also detected at PD under treatment with afatinib. T790M loss was never seen. Among eight patients in whom T790M was not detected just before afatinib treatment, tumor response to afatinib was observed in five patients but afatinib was not effective in three patients in whom afatnib re-challenge was performed. T790M was detected in these three patients at the time of acquired resistance to first afatinib treatment. In total, six of fifteen patients who were negative for T790M (40.0%) turned to positive under afatinib treatment. Loss of 790M was never observed (Fig 2).

We examined EGFR L858R mutation and exon19 deletion with the same samples as those on which we examined T790M, to verify whether ctDNA existed in plasma samples. The result is shown in S3 Table.

### Detection of EGFR C797S mutation, BRAF V600E mutation, and MET amplification

Next, we examined the *EGFR* C797S mutation, *BRAF* V600E mutation, and *MET* amplification to elucidate mechanisms of acquired resistance to afatinib other than those involving T790M. *EGFR* C797S, *BRAF* V600E, and *MET* amplification were not detected in ctDNA from any patients before afatinib treatment. The results of examinations with ctDNA and re-biopsy samples at PD under afatinb are shown in Table 2. *EGFR* C797S mutation was detected in three patients (patients 5, 16, and 18), and all of these mutations were T2389A. In patient 5, C797S was detected in ctDNA by both MBP-QP and ddPCR, but was not detected in a re-biopsy sample from liver. The allele frequency of C797S in ctDNA assessed by ddPCR was 0.19%. In patient 16, re-biopsy samples were obtained from two sites: axillary lymph node and liver. C797S was detected by ddPCR (but was not detected by MBP-QP) in the sample from liver, with allele frequency 0.27%. In patient 18, C797S was detected only in a sample from pleura, again by ddPCR only, with allele frequency 0.032%. The *BRAF* V600E mutation was detected in cdDNA from only one patient (patient 7), with allele frequency 2.49%. *MET* amplification was not detected in any samples obtained at PD under afatinib.

**Table 2:** C797S, V600E, and MET amplification detection with ctDNA and re-biopsy at PD to afatinib.

Patient*	C797S detection in ctDNA		C797S detection in re-biopsy		V600E detection in ctDNA	V600E detection in re-biopsy	*MET* CN ^†^ in ctDNA	*MET* C N ^†^ in re-biopsy
	MBP-QP	ddPCR	MBP-QP	ddPCR	ddPCR	ddPCR	ddPCR	ddPCR
1	Negative	Negative	Negative	Negative	Negative	Negative	0.9	0.7
2	Negative	Negative	NE	NE	Negative	NE	0.9	NE
3	Negative	Negative	NE	NE	Negative	NE	1.0	NE
4	Negative	Negative	NE	NE	Negative	NE	1.0	NE
5	Positive	Positive	Negative	Negative	Negative	Negative	1.1	1.5
6	Negative	Negative	NE	NE	Negative	NE	0.9	NE
7	Negative	Negative	NE	NE	Positive	NE	1.0	NE
8	Negative	Negative	Negative	Negative	Negative	Negative	0.9	0.9
9	Negative	Negative	NE	NE	Negative	NE	0.7	NE
10	Negative	Negative	Negative	Negative	Negative	Negative	0.8	0.9
11	Negative	Negative	Negative	Negative	Negative	Negative	0.9	1.1
12	Negative	Negative	NE	NE	Negative	NE	0.8	NE
13	NE	NE	NE	NE	NE	NE	NE	NE
14	Negative	Negative	Negative	Negative	Negative	Negative	1.0	1.0
15	Negative	Negative	NE	NE	Negative	NE	0.9	NE
16	Negative	Negative	LN:Negative	LN:Negative	Negative	LN:Negative	1.1	LN:1.1
			Liver: Negative	Liver: Positive		Liver: Negative		Liver:0.8
17	Negative	Negative	NE	NE	Negative	NE	NE	NE
18	Negative	Negative	Negative	Positive	Negative	Negative	1.0	1.1
19	Negative	Negative	NE	NE	Negative	NE	0.9	NE
20	NE	NE	NE	NE	NE	NE	NE	NE

*Patients 1–7 were treated with afatinib as first-line treatment or after discontinuation of 1^st^ generation EGFR-TKI due to adverse effect. Patients 8–20 were treated with afatinib after acquired resistance to previous EGFR-TKI, as EGFR-TKI re-challenge.

^†^ *MET* copy number was calculated as the ratio of the concentrations of *MET* and *RPP30*

Abbreviations: PD, progressive disease; MBP-QP, mutation-biased PCR and quenching probe method; ddPCR, droplet digital PCR; CN, copy number; NE, not evaluated; LN, lymph node

### Detection of EGFR T790M and C797S mutation after afatinib treatment and response to osimertinib

**Fig 3:**
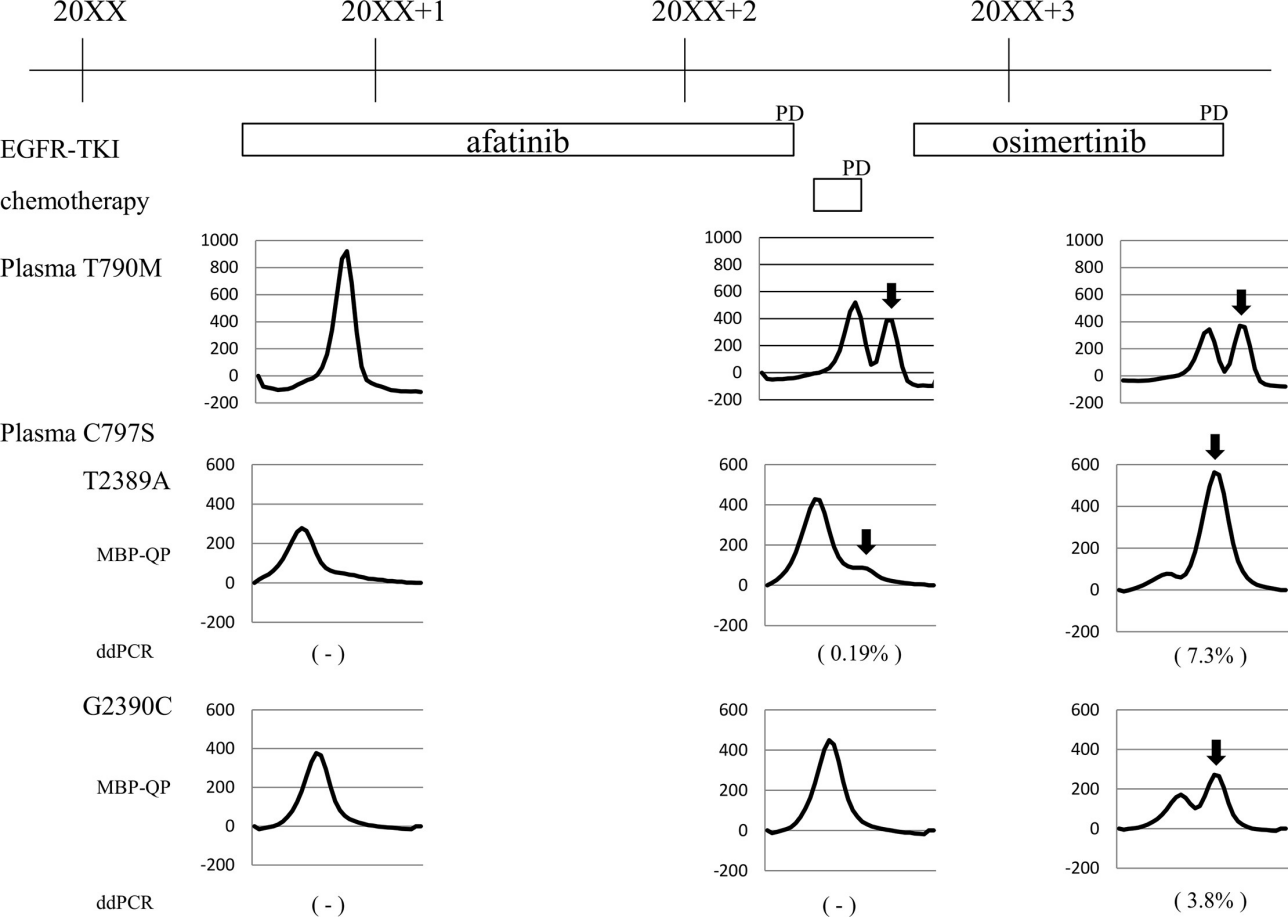
Serial analysis of T790M and C797S mutations in patient 5. Black arrows show mutant peaks by the MBP-QP method. The negative sign (-) indicates undetectable by droplet digital PCR (ddPCR), and the numbers are allele frequencies determined by ddPCR.

Six patients in whom T790M was detected in re-biopsy samples had osimertinib administered as the post-afatinib treatment (Table 3). Although C797S was detected simultaneously with T790M in three of these patients (patients 5, 16, and 18), two of these patients achieved PR under osimertinib. However, the PFS of patients in whom both T790M and C797S were detected was somewhat shorter than that of patients with T790M only. We followed C797S in patients 5 and 18 until PD under osimertinib. In patient 5, C797S was examined with ctDNA. The mutant peak of C797S T2389A detected by MBP-QP increased, and that of C797S G2390C newly appeared (Fig 3). The C797S mutations T2389A and G2390C were confirmed by ddPCR and the allele frequency of C797S T2389A increased from 0.19% to 7.3%. In patient 18, C797S was not detected in ctDNA by either MBP-QP or ddPCR. The C797S T2389A mutation was detected by ddPCR in the pleura and in pleural effusion. The allele frequency of C797S was 0.032% in pleura at PD under afatinib, and it was 0.067% in pleura and 0.059% in pleural effusion at PD under osimertinib, a difference that is not significant. In patient 16, the mutation status differed by site of metastatic lesion: whereas an axillary lymph node metastasis was T790M positive and C797S negative, a liver metastasis was T790M negative and C797S positive. As a result, osimertinib was effective against the axillary lymph node metastasis but did not prevent progression of the liver metastasis.

**Table 3:** T790M and C797S detection at PD to afatinib and effect of osimertinib.

Patient	Detection of T790M		Detection of C797S		Effect of osimertinib	PFS under osimertinib (days)
	ctDNA	Re-biopsy	ctDNA	Re-biopsy		
5	Positive	Positive	Positive	Negative	PR	258
10	Positive	Positive	Negative	Negative	PR	444 (ongoing)
11	Positive	Positive	Negative	Negative	PR	425
14	Negative	Positive	Negative	Negative	PR	483 (ongoing)
16	Positive	LN:Positive	Negative	LN:Negative	PD	38
		Liver:Negative		Liver:Positive		
18	Positive	Positive	Negative	Positive	PR	383

Abbreviations: PD, progressive disease; PR, partial response; PFS, progression-free survival; LN, lymph node

## Discussion

The mechanisms of acquired resistance to the 2^nd^ generation EGFR-TKI afatinib have not previously been sufficiently clarified. Several analyses using an afatinib resistant cell line revealed some potential mechanisms of acquired resistance, such as the *EGFR* mutations T790M, C797S, L792F, and V843I, *MET* amplification, IGF1R and FGFR1 activation, and epithelial-to-mesenchymal transition [13,29–34]. However, analyses using clinical samples from patients who acquired resistance to afatinib are rare. Lung cancer is heterogeneous and the biological characteristics can vary even among metastatic lesions within a patient. Although re-biopsy is a standard approach to evaluate the mechanisms of acquired resistance, biopsy of a single lesion might not reflect the variety of mechanisms of acquired resistance throughout the body [35–39]. ctDNA is more likely to reflect the main mechanism of acquired resistance of tumors throughout the entire body, although the possibility of a false negative is a problem that remains to be solved. Therefore we chose ctDNA as the sample for monitoring molecular events related with acquired resistance to afatinib.

The frequency of T790M at PD under afatinib was reported to be 36–47% in three studies [20–22]. In the present study, six of fifteen patients (40.0%) who were negative for T790M just before afatinib treatment became T790M positive at PD under afatinib. Three of five patients with T790M detected just before afatinib treatment achieved PR or SD, but T790M was also detected in all patients at PD under afatinib treatment. T790M loss was never seen. Additionally, in patients in whom T790M was detected just before afatinib treatment, the area under the mutation peak of T790M by MBP-QP was correlated with T790M allele frequency and increased at PD under afatinib. These results suggest that afatinib has a positive but insufficient effect on T790M positive cancer cells, and it is consistent with data on a cell line harboring T790M [5]. It is also presumed that the influence of T790M on acquired resistance to afatinib is less than with first generation EGFR-TKIs.

C797S, which is known as an *EGFR* mutation related to acquired resistance to osimertinib, was detected in three of twenty patients (15%) at PD under afatinib (Table 2, Fig 3). C797S was observed simultaneously with T790M in all patients; both mutations were newly detected at PD under afatinib in two patients, and C797S was newly detected at PD in a patient in addition to previously detected T790M. Allele frequency of C797S at PD under afatinib was very low in all patients, whereas that of T790M was high at PD or obviously increased after PD. These data suggest that lung cancers carrying the T790M mutation would be more resistant to afatinib than those carrying the C797S mutation. Experiments using cell culture also showed that afatinib induced T790M and C797S mutations during the development of acquired resistance to afatinib, and the IC_50_ of afatinib was lower in cell lines carrying C797S than in those carrying T790M [40]. Two of three patients who were positive for both T790M and C797S achieved PR to osimertinib, but the PFS of these patients was somewhat shorter than that of those with T790M only. In addition, we observed that the allele frequency of C797S increased after disease progression under osimertinib in one patient. From these observations, it seems possible that the existence of C797S caused shorter PFS, and increased C797S clones lead to acquired resistance to osimertinib. Examination of C797S in addition to T790M at PD under afatinib would enable us to better predict post-treatment osimertinib efficacy.

As for mutations other than those of *EGFR*, *BRAF* V600E was detected in one patient, and *MET* amplification at PD under afatinib was detected in no patients in this study. There have been few reports to date of acquired resistance to afatinib involving other than T790M mutations. In a prospective study, *MET* amplification was observed in one among fourteen patients who acquired resistance to afatinib and underwent re-biopsy [22]. It may be rare for acquired resistance to afatinib to occur with EGFR-independent resistance mechanisms, contrary to 1^st^ and 3^rd^ generation EGFR-TKIs, although further examination with comprehensive analysis is needed to clarify this. It was reported in one study that median treatment time on osimertinib post afatinib treatment was 20.2 months [10]; this is considerably longer than the PFS in the AURA3 study (10.1 months), in which 93% of patients were treated with osimertinib after disease progression under 1^st^ generation EGFR-TKI [9]. If it is confirmed by comprehensive analysis that afatinib does not readily induce genetic alterations other than those in *EGFR*, this might explain the longer treatment period of osimertinib after afatinib.

There are several potential limitations of our study. It was retrospective with a small sample size, and the number of investigated molecular markers of acquired resistance to afatinib was not large. In addition, many patients were administered afatinib as EGFR-TKI re-challenge after acquired resistance to first generation EGFR-TKI, so it is difficult to rule out the possibility that mutations detected in this study were influenced by the previous EGFR-TKI treatment. Although T790M is a major mutation related with acquired resistance to EGFR-TKI, its coexistence with other genetic alterations surely affects subsequent treatment. In recent years, next generation sequencing (NGS) has been applied to clarify the mechanisms of acquired resistance. NGS can be used to screen multiple mutations concurrently using limited clinical samples. Furthermore, NGS has been applied with ctDNA and seemed to be suitable for monitoring how mechanisms of acquired resistance change with treatment. Further studies with comprehensive analysis are needed to clarify the mechanisms of acquired resistance to afatinib to facilitate consideration of the best sequence of EGFR-TKI.

## Supporting information

S1 Fig Flow chart of this study. (TIF)

S1 Table Characteristics of patients with lung adenocarcinoma who acquired resistance to afatinib. Abbreviations: EGFR-TKI, epidermal growth factor receptor tyrosine kinase inhibitor; PR, partial response; SD, stable disease; PD, progressive disease.
(DOCX)

S2 Table Responses to previous EGFR-TKI treatment and reasons for discontinuation. Abbreviations: EGFR-TKI, epidermal growth factor receptor tyrosine kinase inhibitor; AE, adverse event; PR, partial response; SD, stable disease; PD, progressive disease; NE, not evaluated.
(DOCX)

S3 Table EGFR T790M and activating mutation detection with ctDNA. Abbreviations: EGFR-TKI, epidermal growth factor receptor tyrosine kinase inhibitor; NE, not evaluated; Exon19 del, EGFR exon19 deletion.
(DOCX)
