# Supplementary figures and images for: Mechanisms of acquired resistance to afatinib clarified with liquid biopsy
Source: PLoS One. 2018 Dec 14;13(12):e0209384. doi: 10.1371/journal.pone.0209384 (PMC6294373; doi:10.1371/journal.pone.0209384)

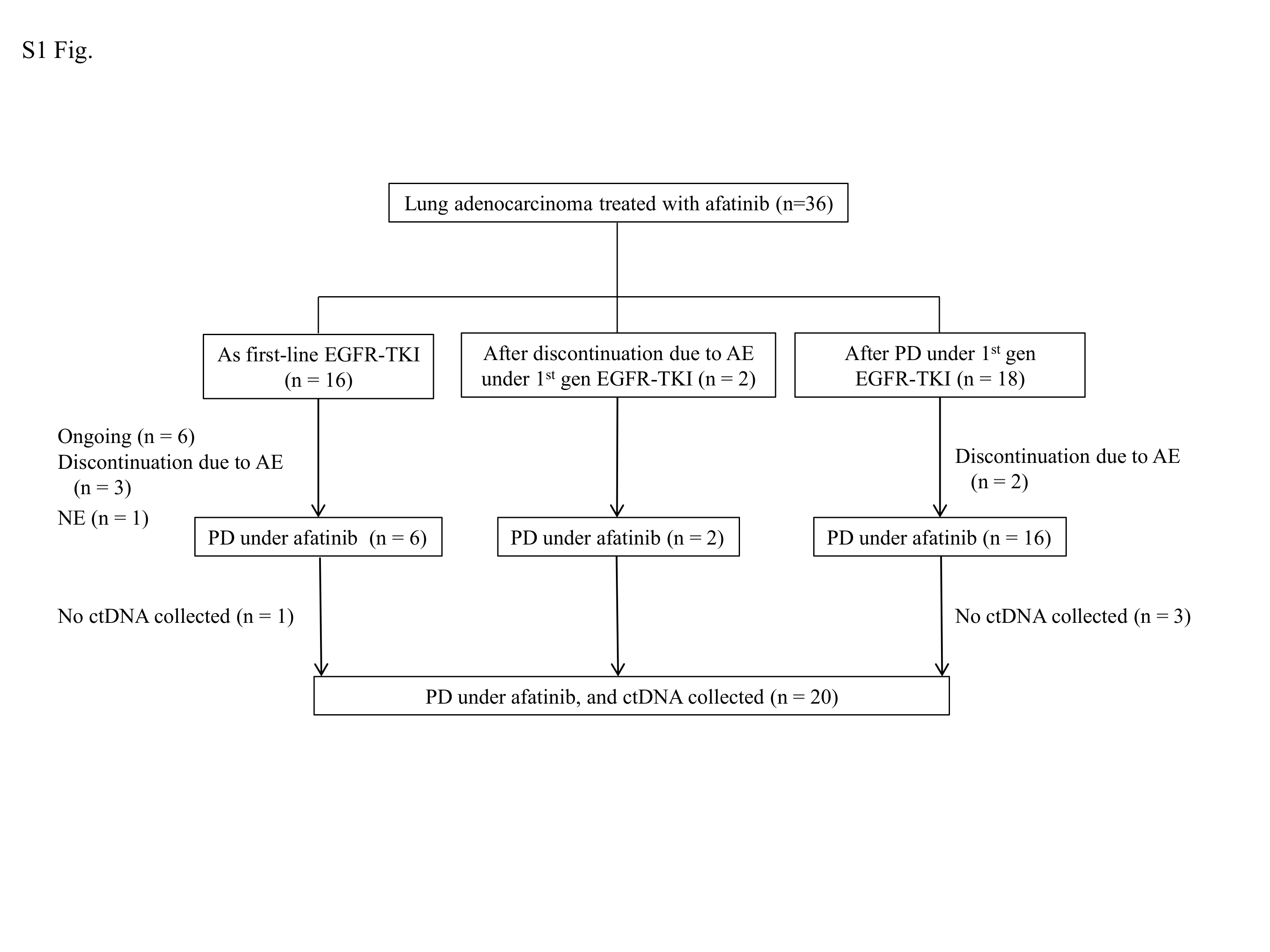

Supplement: S1 Fig — (TIF) [file pone.0209384.s001.tif]
