# Supplementary material for: Mechanisms of acquired resistance to afatinib clarified with liquid biopsy
Source: PLoS One. 2018 Dec 14;13(12):e0209384. doi: 10.1371/journal.pone.0209384 (PMC6294373; doi:10.1371/journal.pone.0209384)
Supplement: S1 Table — Abbreviations: EGFR-TKI, epidermal growth factor receptor tyrosine kinase inhibitor; PR, partial response; SD, stable disease; PD, progressive disease. (DOCX) [file pone.0209384.s002.docx]

**S1 Table.**

**Characteristics of patients with lung adenocarcinoma who acquired resistance to afatinib**

| Total | n=20 |
| --- | --- |
| Age (years old)  Median (years old) | 40-84  63 |
| Sex  Male  Female | 9 (45.0%)  11 (55.0%) |
| Smoking status  Smoker  Never smoker | 9 (45.0%)  11 (55.0%) |
| *EGFR* mutation  Exon19 deletion  L858R  G719A | 8 (40.0%)  11 (55.0%)  1 (5.0%) |
| Number of pre-afatinib EGFR-TKI treatments  0  1  2  3 | 5 (25.0%)  7 (35.0%)  5 (25.0%)  3 (15.0%) |
| Response to afatinib  PR  SD  PD | 6 (30.0%)  9 (45.0%)  5 (25.0%) |

Abbreviations: EGFR-TKI, epidermal growth factor receptor tyrosine kinase inhibitor;

PR, partial response; SD, stable disease; PD, progressive disease
