# Supplementary material for: Mechanisms of acquired resistance to afatinib clarified with liquid biopsy
Source: PLoS One. 2018 Dec 14;13(12):e0209384. doi: 10.1371/journal.pone.0209384 (PMC6294373; doi:10.1371/journal.pone.0209384)
Supplement: S2 Table — Abbreviations: EGFR-TKI, epidermal growth factor receptor tyrosine kinase inhibitor; AE, adverse event; PR, partial response; SD, stable disease; PD, progressive disease; NE, not evaluated. (DOCX) [file pone.0209384.s003.docx]

**S2 Table**

**Responses to previous EGFR-TKI treatment and reasons for treatment discontinuation**

| Patient | Primary EGFR mutation | Previous EGFR-TKI treatment | Response to previous EGFR-TKI | | Reason of discontinuation of treatment |
| --- | --- | --- | --- | --- | --- |
|  |  |  | ORR | Duration of treatment  (days) |  |
| 1 | Exon 19 deletion | Gefitinib | PR | 220 | AE (liver disfunction) |
| 2 | G719A | None | NE | NE |  |
| 3 | Exon 19 deletion | None | NE | NE |  |
| 4 | Exon 19 deletion | None | NE | NE |  |
| 5 | Exon 19 deletion | None | NE | NE |  |
| 6 | Exon 19 deletion | None | NE | NE |  |
| 7 | Exon 19 deletion | Erlotinib | PR | 205 | AE (liver disfunction) |
| 8 | Exon 19 deletion | Gefitinib | PR | 266 | PD |
| 9 | L858R | Gefitinib  Erlotinib | PR  SD | 146  47 | AE (liver disfunction)  PD |
| 10 | L858R | Gefitinib | PR | 475 | PD |
| 11 | L858R | Gefitinib | SD | 918 | PD |
| 12 | L858R | Gefitinib  Erlotinib | PR  SD | 437  144 | PD  PD |
| 13 | L858R | Gefitinib  Erlotinib | NE  PR | 46  336 | AE (liver disfunction)  PD |
| 14 | Exon 19 deletion | Gefitinib | PR | 393 | PD |
| 15 | L858R | Gefitinib  Erlotinib | PR  SD | 358  381 | PD  PD |
| 16 | L858R | Gefitinib | PR | 374 | PD |
| 17 | L858R | Gefitinib  Erlotinib+BEV | PD  SD | 39  176 | PD  PD |
| 18 | L858R | Gefitinib  Erlotinib  Afatinib | NE  PR  SD | 46  336  211 | AE (liver disfunction)  PD  PD |
| 19 | L858R | Gefitinib  Erlotinib  Afatinib | PR  SD  PR | 146  47  206 | AE (liver disfunction)  PD  PD |
| 20 | L858R | Gefitinib  Erlotinib  Afatinib | PR  SD  SD | 437  144  159 | PD  PD  PD |

Abbreviations: EGFR-TKI, epidermal growth factor receptor tyrosine kinase inhibitor; AE, adverse event

PR, partial response; SD, stable disease; PD, progressive disease, NE, not evaluated
