# Supplementary material for: Mechanisms of acquired resistance to afatinib clarified with liquid biopsy
Source: PLoS One. 2018 Dec 14;13(12):e0209384. doi: 10.1371/journal.pone.0209384 (PMC6294373; doi:10.1371/journal.pone.0209384)
Supplement: S3 Table — Abbreviations: EGFR-TKI, epidermal growth factor receptor tyrosine kinase inhibitor; NE, not evaluated; Exon19 del, EGFR exon19 deletion. (DOCX) [file pone.0209384.s004.docx]

**S3 Table.**

**EGFR T790M and activating mutation detection with ctDNA**

| Patient | EGFR mutation status in ctDNA at PD under previous EGFR-TKI | | EGFR mutation status in ctDNA just before afatinib | | EGFR mutation status in ctDNA under afatinib | |
| --- | --- | --- | --- | --- | --- | --- |
|  | T790M | Activating mutation | T790M | Activating mutation | T790M | Activating mutation |
| 1 |  |  | Negative | Positive (Exon19 del) | Negative | Negative (Exon19 del) |
| 2 |  |  | Negative | NE | Negative | NE |
| 3 |  |  | Negative | Negative (Exon19 del) | Negative | Negative (Exon19 del) |
| 4 |  |  | Negative | Negative (Exon19 del) | Negative | Negative (Exon19 del) |
| 5 |  |  | Negative | Negative (Exon19 del) | Negative | Negative (Exon19 del) |
| 6 |  |  | Negative | Positive (Exon19 del) | Negative | Positive (Exon19 del) |
| 7 |  |  | Negative | Positive (Exon19 del) | Positive | Positive (Exon19 del) |
| 8 | Negative | Positive (Exon19 del) | Positive | Negative (Exon19 del) | Positive | Negative(Exon19 del) |
| 9 | Positive | Positive (L858R) | Negative | Positive (L858R) | Positive | Positive (L858R) |
| 10 | NE | NE | Positive | Positive (L858R) | Positive | NE |
| 11 | NE | NE | Positive | Positive (L858R) | Positive | NE |
| 12 | Negative | Positive (L858R) | Negative | Positive (L858R) | Positive | Positive (L858R) |
| 13 | Negative | Negative (L858R) | Negative | Positive (L858R) | Positive | Positive (L858R) |
| 14 | Positive | NE | Negative | Negative (Exon19 del) | Negative | Positive (Exon19 del) |
| 15 | NE | NE | Negative | Positive (L858R) | Negative | Positive (L858R) |
| 16 | Positive | NE | Positive | Positive (L858R) | Positive | Positive (L858R) |
| 17 | NE | NE | Positive | Positive (L858R) | Positive | Positive (L858R) |
| 18 | Positive | Positive (L858R) | Negative | Positive (L858R) | Positive | Positive (L858R) |
| 19 | Positive | Positive (L858R) | Negative | Positive (L858R) | Negative | Positive (L858R) |
| 20 | Positive | Positive (L858R) | Negative | Positive (L858R) | NE | NE |

Abbreviations: EGFR-TKI, epidermal growth factor receptor tyrosine kinase inhibitor; NE, not evaluated,

Exon19 del, EGFR exon19 deletion
